# What are the benefits of therapeutic drug monitoring in the optimization of adalimumab therapy? a systematic review and meta-analysis up to 2022

**DOI:** 10.3389/fphar.2024.1376708

**Published:** 2024-07-08

**Authors:** Yun Li, Cheng Xie, Xiaoliang Ding, Ziyang Wu, Jingjing Zhang, Jianguo Zhu, Liyan Miao

**Affiliations:** ^1^ Department of Pharmacy, The First Affiliated Hospital of Soochow University, Suzhou, China; ^2^ Institute for Interdisciplinary Drug Research and Translational Sciences, Soochow University, Suzhou, China; ^3^ College of Pharmaceutical Sciences, Soochow University, Suzhou, China; ^4^ National Clinical Research Center for Hematologic Diseases, The First Affiliated Hospital of Soochow University, Suzhou, China

**Keywords:** adalimumab, biologics, therapeutic drug monitoring, benefits, optimization

## Abstract

**Aims:**

Persistent uncertainties exist surrounding the therapeutic drug monitoring (TDM) of adalimumab in clinical settings. To address these issues, we conducted a systematic review to assess the current evidence regarding the benefits of TDM for adalimumab.

**Methods:**

PubMed, EMBASE, and Cochrane Databases were searched from inception to October 2022. The trials regarding to the list three key questions were considered: 1) Could routine proactive TDM assist in improving outcomes in patients receiving adalimumab? 2) Could reactive TDM assist in guiding subsequent treatment strategies for patients with treatment failure to adalimumab? 3) Could TDM assist in informing dose reduction or discontinuation in patients with low disease activity or in remission treated with adalimumab? Two reviewers independently selected the studies and extracted the data. Meta-analysis was performed to calculate the relative risk (RR) and 95% confidence interval (CI).

**Results:**

A total of 9 studies was included in this review. For proactive TDM, meta-analysis indicated that proactive TDM (n = 163/257, 63.42%) showed no significant superiority over reactive TDM and/or conventional management (n = 336/606, 55.44%) in achieving and/or maintaining clinical remission by random effects model (RR: 1.24, 95% CI 0.98–1.58, *I* ^2^ = 73%). There were three studies that supporting the reactive TDM, low drug levels in the absence of anti-drug antibodies (ADA) strongly indicate the need for dose intensification, and infliximab is a feasible choice for patients with low drug levels and ADA positivity. While swapping to another class should be considered in patients with adequate drug levels. In addition, TDM can help clinicians optimize dosing schedules and prevent overtreatment in patients who have achieved low disease activity and sufficient drug concentrations, with no predictive value for successful adalimumab discontinuation.

**Conclusion:**

Current evidence suggests that proactive TDM is numerically but not statistically significant superiority over reactive TDM and/or conventional management. Reactive TDM can aid in understanding treatment failure and developing subsequent therapy. For patients reaching low disease activity and remission, TDM can help successful dose reduction, while it cannot inform the successful drug discontinuation. However, existing trials are limited, and more well-designed trials are necessary to clarify the role of TDM in adalimumab treatment.

## 1 Introduction

Adalimumab (ADM), a fully human monoclonal antibody that neutralizes tumor necrosis factor-α (TNF-α), was initially approved for the treatment of moderate to severe rheumatoid arthritis (RA) in 2002 (Rau, 2002). Since then, it has been found to be effective in treating a variety of other conditions, such as ankylosing spondylitis, psoriasis, Crohn’s disease (CD), ulcerative colitis (UC), uveitis, and juvenile idiopathic arthritis, making it the most widely used agent.

Therapeutic drug monitoring (TDM) is a practical method used to monitor the drug concentration and their metabolites in the blood, which can help guide clinical medication decisions, enhance drug effectiveness, prevent drug toxicity, and establish personalized treatment schedules. Recently, TDM has become essential in biological therapy due to the impact of drug concentrations of TNF-α inhibitors on clinical outcomes (Pouw et al., 2015; Rinawi et al., 2021). Anti-drug antibodies (ADA) play a significant role in the inter-individual variability of drug clearance, leading to insufficient drug exposure and treatment failure, such as primary non-response (PNR) and loss-of-response (LOR) (Bartelds et al., 2011; Baert et al., 2016; Ding et al., 2020). Reactive TDM refers to measure biological concentration and ADA in patients experiencing treatment failure. This approach is endorsed by the American Gastroenterological Association and expert consensus statements to understand treatment failure (Feuerstein et al., 2017; Mitrev et al., 2017; Cheifetz et al., 2021; Krieckaert et al., 2023), despite the limited quality of evidence. The supported evidence comes primary from studies involving infliximab therapy. It is not yet clear how many benefits of TDM can bring to the clinical application of ADM. However, the use of proactive TDM, which involves scheduled testing and adjusting dosages to achieve predefined target concentrations, lacks consistent recommendations (Feuerstein et al., 2017; Cheifetz et al., 2021). There are persistent uncertainties surrounding the most effective use of TDM in clinical settings. Specifically, the evidence supporting the use of TDM to guide dose reduction or discontinuation in patients achieving deep remission has not been reviewed.

To systematically review the value of TDM in optimizing ADM therapy, three key questions throughout the entire drug treatment process were considered: 1) Could routine proactive TDM assist in improving outcomes in patients receiving ADM? 2) Could reactive TDM assist in guiding subsequent treatment strategies for patients PNR or LOR to ADM? 3) Could TDM assist in informing dose reduction or discontinuation in patients with low disease activity or in remission treated with ADM?

## 2 Methods

### 2.1 Search strategy

This systematic review and meta-analysis was conducted according to the Preferred Reporting Items for Systematic Reviews and Meta-Analyses (PRISMA) statement (Page et al., 2021). We systematically searched PubMed, EMBASE and Cochrane Database from inception to October 2022 to identify applicable studies. A search strategy was created based on the PICO (Population, Intervention, Comparison, Outcomes) questions. The search terms used were combinations of text-free terms and Medical Subject Headings (MeSH) terms as follows: ADM, therapeutic drug monitoring, therapeutic monitoring, serum concentration monitoring. There were no language or publication date restrictions. The full search terminology was included in the Supplementary Table S1. We also hand-searched trial registries such as ClinicalTrials.gov (https://clinicaltrials.gov) and the World Health Organization (WHO) International Clinical Trials Registry Platform (http://apps.who.int/trialsearch) and reference lists of included trials for completeness.

### 2.2 Selection criteria

Studies published as full manuscripts related to the PICO questions were included. These involved studies assessing: 1) Could routine proactive TDM assist in improving outcomes in patients receiving ADM? 2) Could reactive TDM assist in guiding subsequent treatment strategies for patients PNR or LOR to ADM? 3) Could TDM assist in informing dose reduction or discontinuation in patients with low disease activity or in remission treated with ADM? There were no restrictions on disease types or TDM measurements. Reviews, editorials, guidelines, case reports, and studies that focused only on pharmacokinetics and pharmacodynamics were excluded.

### 2.3 Data extraction

Two reviewers (Yun Li and Cheng Xie) independently assessed studies for possible inclusion by reading titles and/or abstracts, then viewed the full texts of the remaining publications to pick up the ultimately available studies. Data extraction was done by one reviewer (Yun Li), and subsequently cross-checked by the other reviewer (Cheng Xie). Any divergences were discussed or determined by a third investigator (Xiaoliang Ding). Following information was abstracted: the first author and publication year, country, study type, sample size, baseline, patients feature, treatment feature, follow-up time, the clinical outcomes and their definitions.

### 2.4 Quality appraisal

Two reviewers (Yun Li and Cheng Xie) independently evaluated the quality of the studies. Disagreements were resolved through discussion and consultation with the third investigator (Xiaoliang Ding). The risk of bias in the randomized controlled trial (RCT) was evaluated according to the standards developed by the Cochrane Bias Risk Tool (Sterne et al., 2019). The quality of the observational studies was assessed using the Newcastle–Ottawa scale (NOS) (Stang, 2010).

### 2.5 Data analysis

In this systematic review, we conducted a narrative review and utilized meta-analysis when dichotomous outcomes were sufficiently similar across studies, considering the diversity of these focused questions. Both fixed-effect and random-effects model were employed to calculate the relative risk (RR) and 95% confidence interval (CI). Heterogeneity of effect size across the studies was tested using Q statistics at the *p* < 0.10 level of significance. We also calculated the *I* ^2^ statistic with a quantitative measure of inconsistency across the studies. The data were pooled by random-effects model in case significant heterogeneity (Cochran test with *p* < 0.10 or *I* ^2^ > 50%) was found. Otherwise, the fixed-effects model was used. Subgroup analysis, sensitivity analysis, and publication bias analysis were not conducted due to the limited number of included studies. The analysis was carried out using the “meta” package in R (version 4.3.2).

## 3 Results

### 3.1 Search results and characteristics of the included studies

**FIGURE 1:**
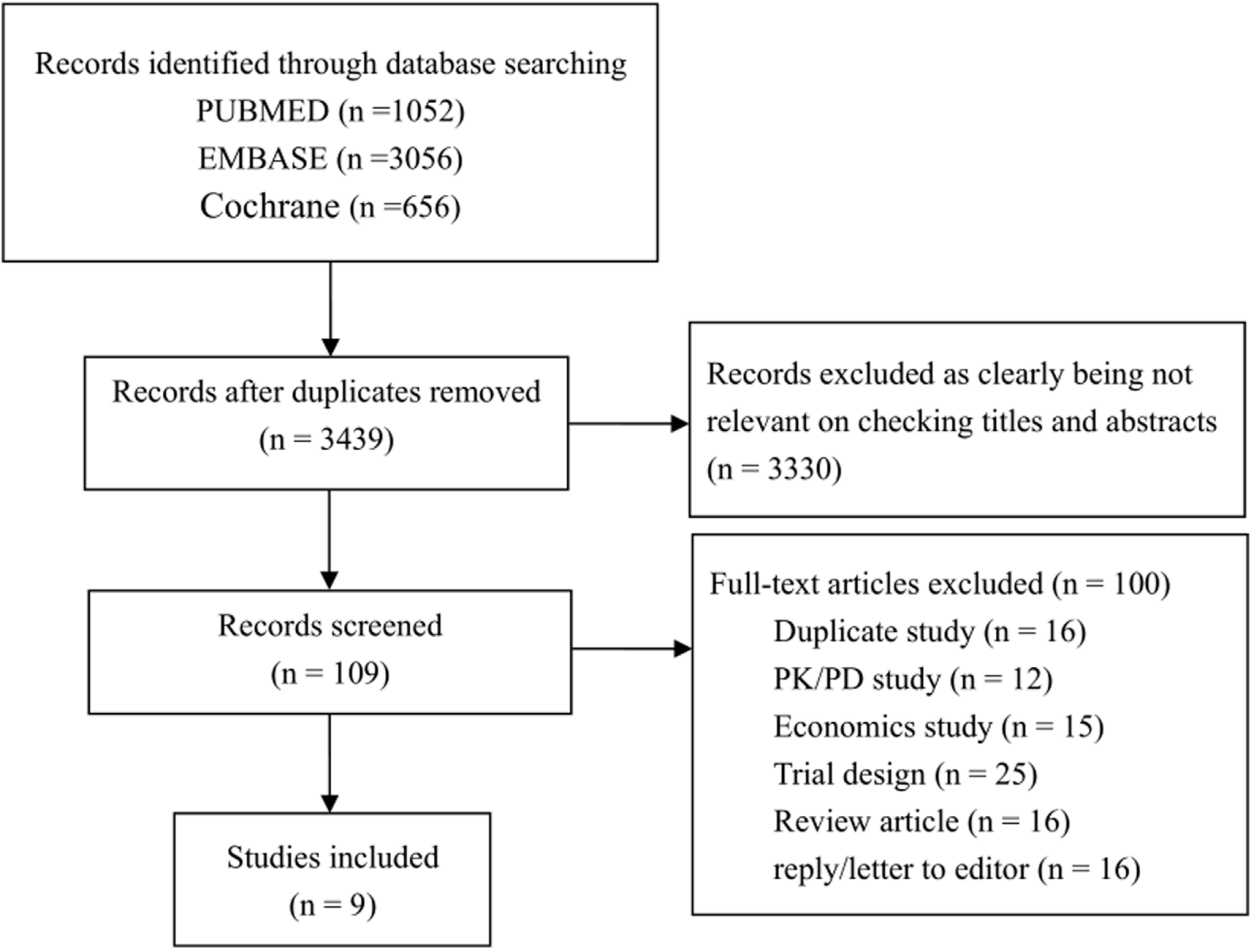
Flow chart depicting the process of selecting the studies included.

Figure 1 shows the research selection process for inclusion in the systematic review. The initial search generated 4764 references. After deleting 1325 duplicate articles titles and abstracts of all the articles were reviewed. A total of 109 studies were reviewed in full, while 100 studies were excluded because of not meet the inclusion criteria. The main reasons for excluding full articles were the inability to extract data related to ADM alone, noncompliance with research objectives, review articles and editorials/letters to editors. The final 9 studies were included (Papamichael et al., 2019; Assa et al., 2019; D’Haens et al., 2022; Panes et al., 2022; Roblin et al., 2014; Roblin et al., 2022; Ulijn et al., 2020; Chen et al., 2016; Lamers-Karnebeek et al., 2019) and the details are shown in Table 1.

**TABLE 1:** Summary of studies.

Trial, author name, year	Study type	Patient population, research duration	Sample size; age (median/range or mean/SD); % males	Phase of study; disease score at inclusion (median/range or mean/SD)	Disease duration; duration of ADA therapy (median/range or mean/SD)	Immunosuppressive treatment (N, %); glucocorticoid treatment (N, %)
Roblin, 2014 (Roblin et al., 2014)	Prospective, cohort, single center (France)	Adult IBD (55% CD), 29 months	82, 43 ± 12, 50	Maintenance phase; CDAI: 340 (110); Mayo: 9 2)	7.4 (3.2) years; 17 9) months	10 (12%); NR
Ulijn, 2020 (Ulijn et al., 2020)	Retrospective, cohort, single center (Netherlands)	Adult RA, 6 months	137, 64.4 ± 13.2, 31.4	Induction phase and maintenance phase; NR	8.7 (12.7) years; 0.75 (3.2) years	Azathioprine: 20 (14.6%); Methotrexate: 60 (43.8%); Leflunomide: 23 (16.8%); Glucocorticoid:24 (17.5%)
Roblin, 2022 (Roblin et al., 2022)	Nonrandomized comparative study, multicenter (2 sites in France)	Adult IBD (70.2% CD), 38 months	131, 36.5 ± 14.6, 50.3	Maintenance phase; CDAI: 300 (240–365); Mayo: 8 (6–10)	80 (32–108) months; 43 (12–68) months	76 (58.0); NR
Chen, 2016 (Chen et al., 2016)	Prospective, cohort, single center (Taiwan)	Adult RA, 24 weeks	64, 55, 9.4	LDA or remission; DAS28: 2.7	9.1 years; 5.8 years	Methotrexate: 58 (90.6%); Salazopyridine: 19 (29.7%); Hydroxychloroquine sulfate: 14 (21.9)
POET, Lamers-Karnebeek 2018 (Lamers-Karnebeek et al., 2019)	Prospective, cohort, multicenter (13 sites in Netherlands)	Adult RA, 12 months	210, 59 ± 13, 31	Stop treatment; DAS28-ESR: 1.96 (0.76)	9 (2.2) years; NR	NR
Papamichael, 2019 (Papamichael et al., 2019)	Retrospective, cohort, multicenter (2 sites in United States)	Adult IBD (81% CD), 37.2 months	382, 25 (19–36), 49	Maintenance phase; NR	9 (3–19) years; NR	Thiopurines: 90 (83%); methotrexate: 19 (17%)
PAILOT, Assa, 2019 (Assa et al., 2019)	RCT, multicenter (9 sites in Israel)	Pediatric CD, 18 months	78, 14 (6–18), 71	Maintenance phase; PCDAI:3.1 (1.0-7.5)	6.0 (1.2-24.7) years; NR	Thiopurines: 28 (35.9%); methotrexate: 7 (9.0%)
SERENE–UC, Panés, 2022 (Panes et al., 2022)	RCT, multicenter (144 sites in 20 countries)	Adult UC, 44 weeks	219, 37 (19–63), 48.6	Maintenance phase; NR	NR	NR
SERENE–CD, D’Haens, 2022 (D’Haens et al., 2022)	RCT, multicenter (93 sites in 19 countries)	Adult CD, 44 weeks	184, 34 (18–73), 53.3	Maintenance phase; CDAI: 303.4 (56.3)	6.4 (8.2) years; NR	25 (27.2); 56 (60.9)

Inflammatory bowel disease, IBD; Crohn’s disease, CD; Crohn’s disease activity index, CDAI; ulcerative colitis, UC; rheumatoid arthritis, RA; not reported, NR; low disease activity, LDA; disease activity score 28, DAS28; erythrocyte sedimentation rate, ESR.

### 3.2 Quality of the included studies

**FIGURE 2:**
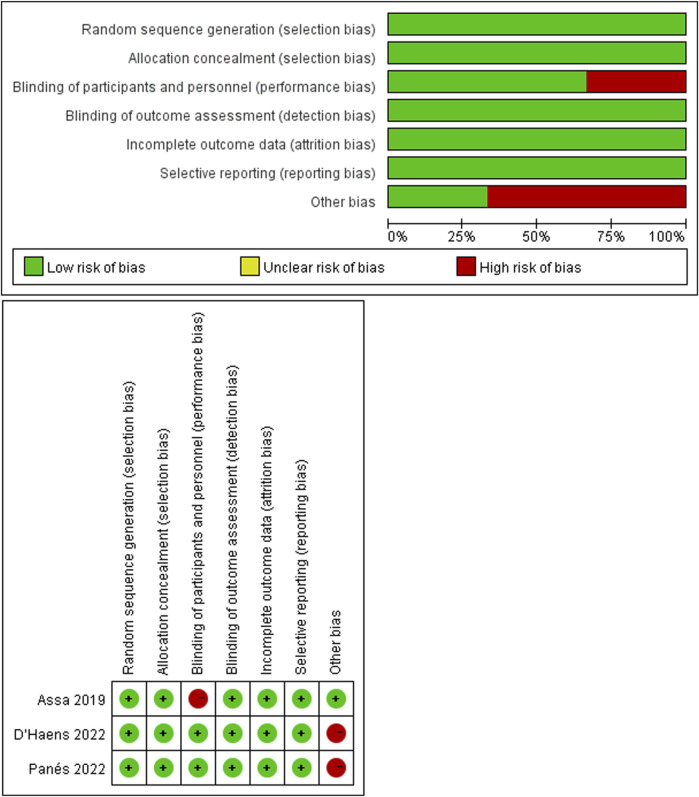
The risk of bias in randomized controlled trials was assessed using the Cochrane risk of bias tool.

A summary of the bias risk data is shown in Figure 2 and Table 2. The quality evaluation of the RCTs revealed that three trials were at high risk of bias across one domain (randomization domain, PAILOT; Other bias, SERENE UC and SERENE CD). PAILOT study is an Open-label study, and most outcomes likely to be influenced. There was no sample size calculation for the maintenance study in SERENE UC and SERENE CD studies. All six observational studies received 8–9 stars out of 9 on the NOS, indicating low risk of bias. Four studies did not fully meet the scoring criteria in terms of inter group comparability and population representativeness. In Roblin’s study, they combined CD and UC together, which may affect the comparability of the results (Roblin et al., 2014; Roblin et al., 2022). In addition, therapeutic groups were not fully comparable at baseline, especially in terms of disease (Roblin et al., 2022). In Lamers Karnebeek’s study, the included population had a longer duration of disease (average of 9 years), which may not fully represent the population of patients with RA (Lamers-Karnebeek et al., 2019). In Papamichael’s research the control group received standard of care which was defined as empirical dose escalation and/or reactive TDM. Therefore, it is not possible to draw clear conclusions between proactive TDM and reactive TDM, as well as between proactive TDM and empirical dose escalation (Papamichael et al., 2019).

**TABLE 2:** Risk of bias in nonrandomized studies using the Newcastle–Ottawa scale.

References	Quality indicators								Total number of stars (out of 9)
	1^a^	2^b^	3^c^	4^d^	5^e^	6^f^	7^g^	8^h^	
Roblin, 2014 (Roblin et al., 2014)	★	★	★	★	★	★	★	★	8
Ulijn, 2020 (Ulijn et al., 2020)	★	★	★	★	★★	★	★	★	9
Roblin, 2022 (Roblin et al., 2022)	★	★	★	★	★	★	★	★	8
Chen, 2016 (Chen et al., 2016)	★	★	★	★	★★	★	★	★	9
Lamers-Karnebeek 2018 (Lamers-Karnebeek et al., 2019)		★	★	★	★★	★	★	★	8
Papamichael, 2019 (Papamichael et al., 2019)	★	★	★	★	★	★	★	★	8

^a^ Indicates exposed cohort truly representative.

^b^ Non exposed cohort drawn from the same community.

^c^ Ascertainment of exposure from a secure record.

^d^ Outcome of interest not present at start of study.

^e^ Study controls for important confounder 1 ± additional confounders.

^f^ Assessment of outcome of record linkage or independent blind assessment.

^g^ Follow-up long enough for outcomes to occur.

^h^ Follow-up adequacy.

### 3.3 Benefits gained from TDM

#### 3.3.1 Scenario A: value of target concentration intervention

**FIGURE 3:**
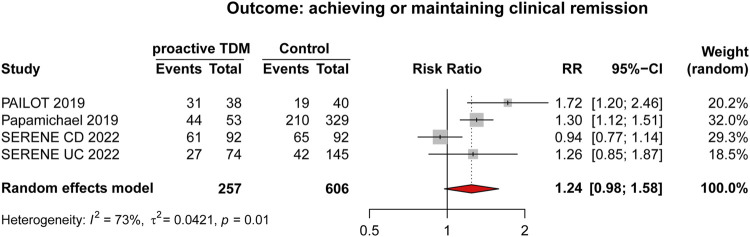
Forest plot comparing proactive TDM vs. control (reactive TDM and/or conventional management which is defined as empirical dose escalation), outcome (achieving or maintaining clinical remission).

Dosage adjustment to target and maintain a predefined drug concentration was the primary format of TDM, specifically referred to routine proactive TDM. This scenario included three RCTs (Assa et al., 2019; D’Haens et al., 2022; Panes et al., 2022) and one observational study (Papamichael et al., 2019), with detailed characteristics outlined in Table 3. Results from the meta-analysis indicated that proactive TDM (n = 163/257, 63.42%) showed no significant superiority over reactive TDM and/or conventional management (n = 336/606, 55.44%) in achieving and/or maintaining clinical remission by random effects model (RR: 1.24, 95% CI 0.98–1.58, *I* ^2^ = 73%; Figure 3).

**TABLE 3:** Clinical studies on the benefits of TDM with ADM.

Study	Population	Primary outcome	Comparison (exposure/intervention)	Results
**Scenario A: Value of target concentration intervention**				
PAILOT, Assa, 2019 (Assa et al., 2019)	Pediatric CD patients responded to ADM induction therapy	Sustained corticosteroid-free clinical remission (PCDAI<10) 18 months	Intervention (proactive TDM, N = 38): ADM was intensified in patients with DL < 5 μg/mL regardless of disease activity Comparator (reactive TDM, N = 40): ADM was intensified only in patients with LOR and DL < 5 μg/mL simultaneously	**Corticosteroid-free clinical remission at all visits** Proactive TDM: 82% (31/38) Reactive TDM: 48% (19/40) Proactive TDM is superior to reactive TDM, resulting in higher corticosteroid-free sustained remission
Papamichael, 2019 (Papamichael et al., 2019)	Adult IBD patients who received maintenance ADM therapy	Treatment failure (LOR or SAE or need for an IBD-related surgery) 3.1 years (median)	Intervention (proactive TDM, N = 53): titrating ADM to concentration typically >10 μg/mL Comparator (standard care, N = 329): empiric dose escalation and/or reactive TDM	**People had treatment failure** Proactive TDM: 17% (7/53) Standard care: 36% (119/329) Proactive TDM may be associated with a lower risk of treatment failure compared to standard care
SERENE–CD, D’Haens, 2022 (D’Haens et al., 2022)	Adult CD patients who achieved clinical response at week 12	Clinical remission (CDAI<150) 44 weeks	Intervention (TDM, N = 92): achieve DL > 5 μg/mL and not exceeding 20 μg/mL Comparator (clinically adjusted, N = 92): dose adjustment based on disease activity	**Achieved clinical remission at week 56** TDM: 66.3% (61/92) Clinically adjusted: 70.7% (65/92) Dose adjustment based primarily on DL did not provide additional clinical benefit over clinical adjustment based on symptoms and biomarkers
SERENE–UC, Panés, 2022 (Panes et al., 2022)	Adult UC patients who achieved clinical response at week 8	Clinical remission (full Mayo score ≤2 with no subscore >1) 44 weeks	Intervention (TDM, N = 92): achieve DL ≥ 10 μg/mL Comparator (40 mg ew, N = 152 or 40 mg eow, N = 145)	**Clinical remission at week 52** TDM: 36.5% (27/74) 40 mg ew: 39.5% (60/152) 40 mg eow: 29.0% (42/145) The efficacy of TDM group was comparable to that of standard dose or high dose group
**Scenario B: Value of guiding treatment strategy optimization in patients experiencing treatment failure**				
Roblin, 2014 (Roblin et al., 2014)	Adult IBD patients, who experienced LOR with 40 mg eow and subsequently receive dosage optimization of 40 mg ew	Clinical remission (CD: CDAI<150 and fecal calprotectin <250 μg/g stool, UC: total Mayo score<3 and endoscopic subscore≤1) 6 months	Three groups defined according to DL and ADA status at LOR Group A (N = 41): DL > 4.9 μg/mL Group B (N = 24): DL < 4.9 μg/mL and ADA negative Group C (N = 17): DL < 4.9 μg/mL and ADA positive	**Proportion of clinical remission** Group A: 29.2% (12/41) Group B: 67% (16/24) Group C: 12% (2/17) Dosage optimization should be considered in patients with low DL and ADA negative
Roblin, 2014 (Roblin et al., 2014)	Adult IBD patients who did not respond to ADM 40 mg ew and subsequently received IFX treatment	Clinical remission (CD: CDAI<150 and fecal calprotectin <250 μg/g stool, UC: total Mayo score<3 and endoscopic subscore≤1) 6 months	Three groups defined according to DL and ADA status at LOR Group A (N = 41): DL > 4.9 μg/mL Group B (N = 24): DL < 4.9 μg/mL and ADA negative Group C (N = 17): DL < 4.9 μg/mL and ADA positive	**Proportion of clinical remission** Group A: 6.9% (2/29) Group B: 25% (2/8) Group C: 80% (12/15) Switch to IFX should be considered in patients with low DL and ADA positive
Roblin, 2022 (Roblin et al., 2022)	Adult IBD patients who experienced LOR with 40 mg eow and DL > 4.9 μg/mL	Therapeutic discontinuation (CD: CDAI>220 and fecal calprotectin >250 μg/g stool, UC: total Mayo score>6 and endoscopic subscore>1, or intolerance to treatment) 24 months	Two strategies according to physician’s decision Optimization group (N = 61): ADM 40 mg ew Swap group (N = 70): switching to UST or VDZ	**Proportion of therapeutic discontinuation** Optimization group: 59.6% (36/61) Swap group: 14.8% (11/70) Swapping to another class is better than dosage optimization in patients who experienced LOR and DL > 4.9 μg/mL
Ulijn, 2020 (Ulijn et al., 2020)	RA patients who experienced inefficacy or toxicity with ADM 40 mg eow and subsequently received another TNFi	EULAR response (DAS28-CRP/ESR, change from baseline ≥1.2 and current DAS28-ESR<3.2 and DAS28-CRP<2.9) 3–6 months	Two groups defined according to DL or ADA status between ≥8 weeks after start ADM treatment and ≤2 weeks after ADM discontinuation DL ≥ 5 μg/mL (N = 17) DL < 5 μg/mL (N = 18) or ADA positive (N = 18) ADA negative (N = 39)	**Proportion of EULAR response** DL ≥ 5 μg/mL: 24% (4/17) DL < 5 μg/mL: 22% (4/18) or ADA positive: 44% (8/18) ADA negative: 44% (17/39) No predictive value of DL or ADA for response to second TNFi
Ulijn, 2020 (Ulijn et al., 2020)	RA patients who experienced inefficacy or toxicity with 40 mg eow and subsequently received non-TNFi treatment	EULAR response (DAS28-CRP/ESR, change from baseline ≥1.2 and current DAS28-ESR<3.2 and DAS28-CRP<2.9) 3–6 months	Two groups defined according to DL or ADA status at stopping ADM treatment DL ≥ 5 μg/mL (N = 18) DL < 5 μg/mL (N = 39) or ADA positive (N = 28) ADA negative (N = 62)	**Proportion of EULAR response** DL ≥ 5 μg/mL: 44% (8/18) DL < 5 μg/mL: 44% (17/39) or ADA positive: 43% (12/28) ADA negative: 39% (24/62) No predictive value of DL or ADA for response to non-TNFi
**Scenario C: Value of guiding dose reduction or discontinuation**				
Chen, 2016 (Chen et al., 2016)	Adult RA patients who had already achieved LDA (DAS28 < 3.2) or remission, switched to ADM dose-halving (40 mg monthly) and a concomitant stable dose of MTX	Persistent remission (DAS28 < 2.6) or persistent LDA (DAS28 < 3.2) 24 weeks	At baseline, 25 and 39 patients had achieved remission and LDA. After 24 weeks of dose-halving, 23 patients were persistent remission and 2 patients turned to LDA, persistent LDA in 24 and disease flare in 15	**The optimal cutoff at baseline for predicting persistent remission or LDA after 24 weeks of dose-halving:** persistent remission: 6.4 μg/mL (AUC 0.998, *p* < 0.001) persistent LDA:1.9 μg/mL (AUC 0.995, *p* < 0.001)
POET, Lamers-Karnebeek 2018 (Lamers-Karnebeek et al., 2019)	Adult RA patients been using ADM (40 mg every other week) for >1 year and had LDA (DAS28 < 3.2, or the rheumatologist’s assessment of LDA with CRP <10 mg/L) for at least 6 months, stopped ADM treatment	Disease flare (>0.6 points increase of DAS28-ESR from baseline, with DAS28-ESR ≥3.2)12 months	Two groups defined according to DL at stopping point DL ≥ 5 μg/mL (N = 117) DL < 5 μg/mL (N = 93)	**Proportion of disease flare** DL ≥ 5 μg/mL: 53% (62/117) DL < 5 μg/mL: 47% (44/93) There is no predictive value of DL for flare risk after stopping ADM treatment

Inflammatory bowel disease, IBD; Crohn’s disease, CD; Crohn’s disease activity index, CDAI; ulcerative colitis, UC; rheumatoid arthritis, RA; therapeutic drug monitoring, TDM; loss-of-response, LOR; serious adverse event, SAE; drug level, DL; every week ew; every other week, eow; infliximab, IFX; ustekinumab, UST; vedolizumab, VDZ; tumour necrosis factor inhibitor, TNFi; methotrexate, MTX; low disease activity, LDA; european league against rheumatism, EULAR; C reactive protein, CRP; erythrocyte sedimentation rate, ESR; anti-dug antibodies, ADA.

#### 3.3.2 Scenario B: value of guiding treatment strategy in patients experiencing treatment failure

Reactive TDM plays a crucial role in understanding and addressing treatment failure with ADM treatment. A total of three studies were included in this scenario and the detailed characteristics were shown in Table 3. Two retrospective cohorts (Roblin et al., 2014; Ulijn et al., 2020) were conducted to evaluate the predictive value of TDM in guiding subsequent strategies. Roblin et al. (2014) studied 82 patients with inflammatory bowel disease (IBD) who experienced disease relapse and were treated with ADM at a weekly dose of 40 mg. Results showed that after 6 months, patients with drug level <4.9 μg/mL and negative ADA tested at time of relapse had a higher clinical remission rate (67%, n = 16/24) compared to those with drug level >4.9 μg/mL (29.2%, n = 12/41) or drug level <4.9 μg/mL and ADA positive (12%, n = 2/17). Subsequently, the remaining fifty-two patients who did not respond to ADM were switched to infliximab treatment. Among these patients, those with drug level <4.9 μg/mL and ADA positive exhibited higher clinical response rate (80%, n = 12/15) than those with drug level >4.9 μg/mL (6.9%, n = 2/29) or drug level <4.9 μg/mL and ADA negative (25%, n = 2/8). Ulijn et al. (Ulijn et al., 2020) conducted a retrospectively study involving 137 RA patients who failed treatment with ADM. The study analyzed the predictive value of TDM results for the use of subsequent biological agents and did not find clear predictive value of ADM concentrations or ADA status in either the TNF-α inhibitors or non-TNF-α inhibitors groups. A nonrandomized controlled trial conducted by Roblin et al. (Roblin et al., 2022) compared dose intensification (n = 61) with swapping to different class (ustekinumab or vedolizumab, n = 70) in patients under ADM maintenance therapy who experienced LOR and had ADM concentration >4.9 μg/mL. The median time without discontinuation in the swapping group was significantly longer than that in the intensification group (24 months vs. 13.3 months, *p* < 0.001). In summary, reactive TDM may assist in understanding the mechanisms of treatment failure and making subsequent treatment strategies. Low drug levels in the absence of ADA strongly indicate the need for dose intensification, with infliximab being a viable option for patients with low drug levels and ADA positive. While swapping to another class should be considered in patients with adequate drug levels.

#### 3.3.3 Scenario C: value of guiding dose reduction or discontinuation

TDM can help reduce overtreatment in patients with low disease activity or in remission by identifying higher drug concentrations. This approach allows for dose reduction or tapering while still maintaining efficacy. Two studies (Chen et al., 2016; Lamers-Karnebeek et al., 2019) were included in this scenario, and their characteristics were shown in Table 3. Chen et al. (2016) evaluated the predictive value of ADM concentrations for dose reduction. 64 RA patients who had already achieved low disease activity (LDA) or remission after receiving ADM full-dose therapy at least 2 years were included, and then received ADM dose-halving at a dose of 40 mg monthly. After 24-week follow-up, they found that ADM concentration above a cutoff of 6.4 μg/mL predicted a persistent remission (AUC: 0.998, 95% CI: 0.936-1.000, sensitivity: 100%, specificity: 93.4%), and a persistent LDA (AUC: 0.995, 95% CI: 0.931-1.000, sensitivity: 93.9%, specificity: 100%) after dose halving. ADM dose halving is feasible for patients who have achieved remission and adequate drug levels. Lamers-Karnebeek et al. (2019) investigated whether the ADM concentration and ADA status predict disease flares after ADM cessation in RA patients who received ADM therapy for more than 1 year and achieved LDA for at least 6 months. 210 RA patients with 1 year follow-up after ADM discontinuation were included and analyzed. 62 (53%) of 117 patients with ADM concentrations ≥5 μg/mL experienced a flare versus 44 (47%) of 93 patients with concentrations <5 μg/mL, with no cut-off of ADM concentration at stopping ADM clearly predicted disease flare. TDM can help clinicians optimize dosing schedules and prevent overtreatment in patients who have achieved LDA and sufficient drug concentrations, with no predictive value for successful ADM discontinuation.

## 4 Discussion

**FIGURE 4:**
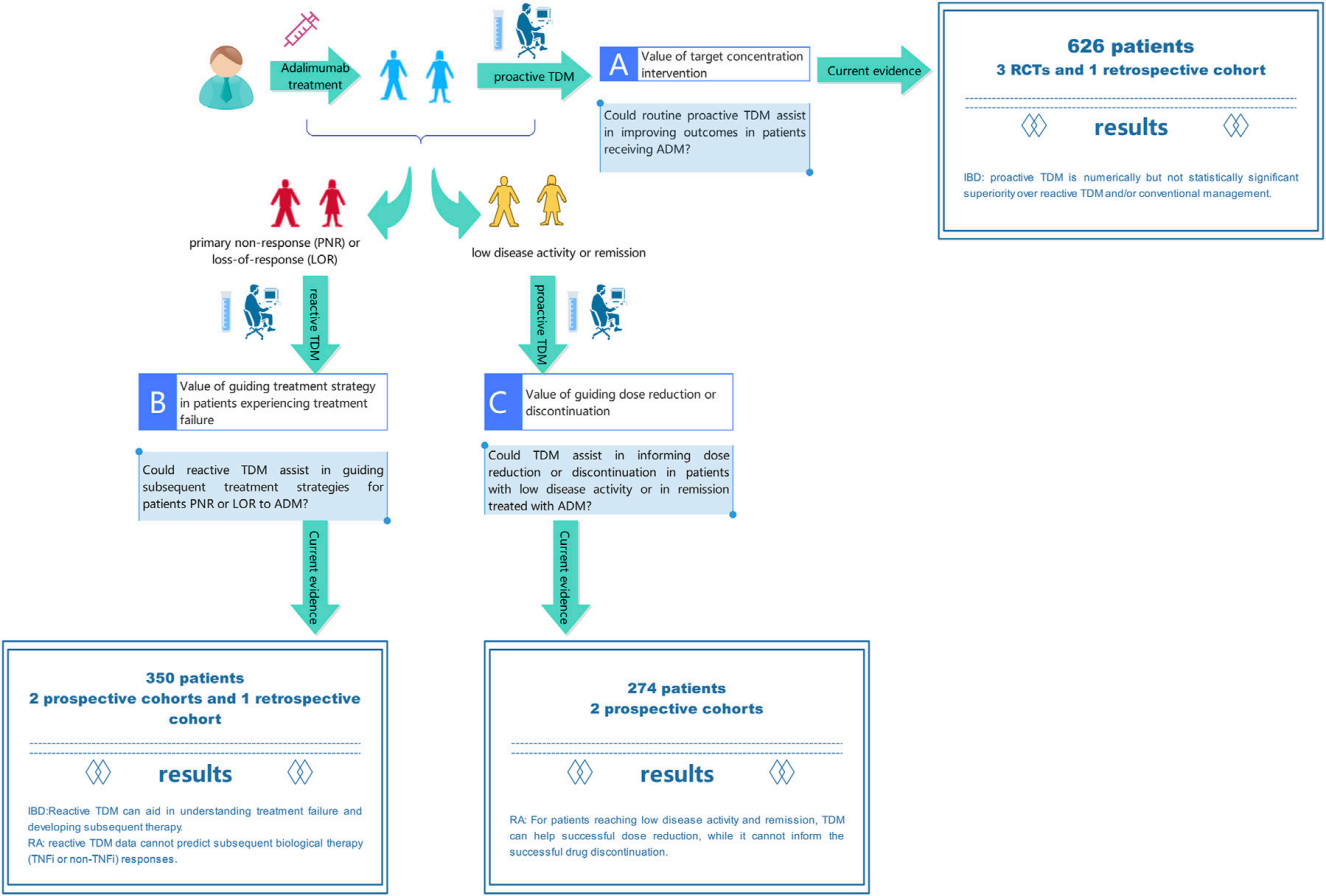
Current evidence on benefits of therapeutic drug monitoring throughout the entire process of adalimumab treatment.

In clinical setting, TDM typically involves adjusting the dosage based on blood concentrations and using pharmacometrics model to ensure that the concentration falls within the desired range to achieve optimal efficacy and avoid adverse reaction. The clinical implementation of TDM of ADM is intricate, mainly due to the need to adjust treatment plans based on different clinical scenarios and TDM results. Our study outlines the benefits of TDM in the entire clinical process of ADM treatment for various diseases. The comprehensive clinical scenarios and evidence are demonstrated in Figure 4.

In scenario A, people hope to obtain the drug concentration and antibody level of ADM to actively intervene and achieve better therapeutic effects. In a meta-analysis of 3 RCTs and 1 retrospective cohort involving 626 IBD patients treated with ADM. Numerically but not statistically significant superiority of proactive TDM over reactive TDM and/or conventional management in achieving and/or maintaining clinical remission was observed. Our results are in line with previous studies on TNF-α inhibitors (Nguyen et al., 2022) which included 9 RCTs (6 for infliximab and 3 for ADM) in patients with IBD. There was no significant difference in the risk of failing to maintain clinical remission in patients who underwent proactive TDM vs. conventional management. Disease duration, concomitant immunomodulators, disease activity at baseline, and optimization of therapy before randomization did not modify this association. Exposure response relationship studies in IBD patients clearly demonstrate that higher anti-TNF drug concentrations are associated with clinical, biochemical, endoscopic, and histological remission (Zittan et al., 2016; Ward et al., 2017; Papamichael et al., 2018). According to reports, proactive TDM is important not only during maintenance therapy but also during induction therapy. Research has shown that ADA can develop as early as the second week in CD patients, leading to unresponsiveness. Proactive TDM can detect low concentrations at the fourth week to avoid immunogenicity and impact patient prognosis (Ungar et al., 2016). However, it seems that we have not obtained the expected evidence of benefits of ADM proactive TDM, but it is worth noting that the included literatures varied in study design, with moderate heterogeneity. The results may be influenced by factors such as patient population, sample size, study time, and detection method, etc. Therefore, more high-quality research is needed to provide additional evidence to clarify benefits of proactive TDM. Proactive TDM may be more important in more severely active patients and those with higher drug clearance rates, such as during induction therapy and in patients with acute severe UC and severe CD. These patients have a high burden of inflammation, increased drug clearance rates, and therefore a higher risk of insufficient drug exposure, immunogenicity, and treatment failure (Brandse et al., 2015; Brandse et al., 2016; Ungar et al., 2016; Battat et al., 2021). Another population with high drug clearance rates is the pediatric population (Jongsma et al., 2020; Winter et al., 2020). Assa et al. conducted relevant studies on pediatric IBD patients and demonstrated that proactive TDM can guide higher frequency treatment strategy adjustments, resulting in higher sustained response rates in the absence of corticosteroids and biological responses (Assa et al., 2019).

In scenario B, reactive TDM is performed when the patient experiencing treatment failure (Krieckaert et al., 2015; Irving and Gecse, 2022; Papamichael et al., 2022). For example, approximately one-third of IBD patients do not respond to TNF-α inhibitors treatment, and among those who initially respond, the LOR is an important clinical issue. In the first year of treatment, up to 40% of patients experience this condition (Colombel et al., 2007). For unresponsive patients, empirical dose escalation therapy may incur significant additional costs, leading to potential ineffective treatment and delaying more effective treatment. In addition, in patients with immune-mediated pharmacokinetic failure (for which ADA was established), additional drug exposure may lead to hypersensitivity reactions. Similarly, excessive drug exposure can lead to a higher risk of drug-related adverse events (such as severe infections). Roblin’s two studies confirmed that different levels of drug and ADA in the IBD population are associated with corresponding treatment adjustment strategies (Roblin et al., 2014; Roblin et al., 2022). Although there was no RCTs to demonstrate superior clinical outcomes of reactive TDM compared to empirical care, the use of TDM can elucidate the mechanism of LOR, whether the lack of response is caused by pharmacokinetic issues, insufficient drug levels, or pharmacological issues of ineffective ADA. TDM provides information for clinical decision-making in unresponsive patients and has intuitive benefits, such as preventing ineffective and potentially dangerous dose escalation in high-titer ADA patients. These results lay the foundation for the guiding the role of TDM in clinical practice and have been introduced in clinical guidelines and expert consensuses to support reactive TDM in ADM treatment (Feuerstein et al., 2017; Khan et al., 2019; Cheifetz et al., 2021; Krieckaert et al., 2023). In addition, Ulijn et al. conducted a study on RA and reported that reactive TDM data cannot predict subsequent biological therapy (TNF-α inhibitors or a non TNF-α inhibitors) responses in patients who failed treatment with ADM (Ulijn et al., 2020). On this issue, current researches have not reached a consistent convincing conclusion. In previous studies, it has been suggested that the measurement of ADM serum levels and/or ADA might be helpful for channeling the right patients to a TNF-α inhibitors or a non TNF-α inhibitors, thus increasing overall response chances (Bartelds et al., 2010; Jamnitski et al., 2011; Plasencia et al., 2013). There may be several reasons for these different results. In Ulijn’s study, samples were not collected at the trough level but were randomly collected after injection of ADM. This might have reduced the association between ADA and response. Second, as this was a retrospective study, serum samples and clinical results were not always available, which may have led to selection bias. In summary, further prospective studies with larger sample sizes are needed to confirm whether drug and ADA levels indeed cannot predict disease activity.

In scenario C, due to the considerable interindividual variability in ADM concentrations and the existing exposure-response relationship, a considerable number of patients may experience overtreatment, leading to a higher risk of infection and increased costs. It is crucial in clinical practice to taper the dose to the lowest effective level, considering cost-effectiveness and potential adverse reactions. For patients who have achieved remission, sufficient ADM concentrations (≥6.4 μg/mL) can support successful ADM dose reduction (halving the dose to 40 mg monthly) (Chen et al., 2016). This approach has been validated by a RCT (l’Ami et al., 2018), RA patients with ADM concentrations >8 μg/mL can potentially prolong dosing interval to once every 3 weeks without loss of disease control, leading to reduced drug costs. While other biomarkers, involving patient, treatment, disease activity, and laboratory and imaging measurements, have not shown predictive value for successful dose reduction (Tweehuysen et al., 2017). It is hypothesized that patients who have achieved LDA and have undetectable drug concentrations may be considered for discontinuation of ADM, as the maintenance of LDA may be independent of the drug. However, data from the POET study (Chen et al., 2016) revealed that a significant proportion of patients (48%) experienced disease flare even with low or undetectable ADM concentrations, indicating that drug concentrations alone may not be sufficient to guide discontinuation decisions. Alternative strategies, such as disease activity-guided dose reduction and withdrawal or step-down approaches, may also be worth considering (van Herwaarden et al., 2015; Fautrel et al., 2016).

Our systematic review and meta-analysis summarized the benefits of TDM in the entire clinical process of ADM treatment for various diseases. However, there are some limitations to consider. In terms of data sources, limitations in data collection methods or sources may affect the reliability and universality of research results. Grey literature, as an important source of information, plays an indispensable role in literature search. Unlike traditional commercial publications, gray literature is usually published by institutions, enterprises, government agencies, professional conferences, and individuals. Its uniqueness makes it important, such as providing comprehensive information, reflecting practical experience and policy advocacy, timely grasping the latest research results, and eliminating publication bias. However, in our study, we only manually searched the trial registry and the list of references included in the trial, which made our search for grey literature incomplete and needed improvement in future research. Secondly, the included literatures varied in study design and quality. The results may be influenced by factors such as patient population, sample size, study time, and experimental environment, etc. as we excluded studies for which we were unable to extract individual ADM data; consequently, studies related to certain diseases, such as psoriasis and ankylosing spondylitis, were not included. Although evidence of benefits, including CD, UC, and RA, was ultimately included, the patient population, research perspectives, and outcome indicators of these studies were not the same, making it difficult to quantitatively summarize and perform meta-analyses for all literature results. Thirdly, it should be noted that assays used in TDM are varied and not yet standardized and may explain the deviation in results from different studies. Finally, our results are mainly based on the Western population, which means that it is difficult to generalize globally. However, within the scope of the currently published research, this article provides the latest results on the benefits of TDM in the entire process of clinical use and management of ADM.

## 5 Conclusion

The systematic review highlights the current evidence of TDM in ADM treatment. We addressed three clinical concerns regarding the benefits of TDM throughout the ADM treatment process. Current evidence suggests that proactive TDM is numerically but not statistically significant superiority over reactive TDM and/or conventional management in achieving and/or maintaining clinical remission. For patients experiencing treatment failure, reactive TDM can aid in understanding the reasons for treatment failure and developing subsequent treatment schedule. For patients reaching LDA or remission, monitoring drug concentrations can help identify and reduce overtreatment, while it cannot inform the successful drug discontinuation. Evidence was observed across various populations, including those with CD, UC, and RA. They encompass optimizing treatment strategies, enhancing clinical outcomes, improving drug utilization, and reducing treatment costs. However, existing clinical trials are limited and of varying quality. More well-designed, high-quality clinical studies are needed to clarify the role of TDM in different clinical settings.

## Data Availability

The original contributions presented in the study are included in the article/Supplementary Material, further inquiries can be directed to the corresponding authors.

## References

[B1] Assa A. Matar M. Turner D. Broide E. Weiss B. Ledder O. (2019). Proactive monitoring of adalimumab trough concentration associated with increased clinical remission in children with Crohn's disease compared with reactive monitoring. Gastroenterology 157 (4), 985–996. 10.1053/j.gastro.2019.06.003 31194979

[B2] Baert F. Kondragunta V. Lockton S. Vande Casteele N. Hauenstein S. Singh S. (2016). Antibodies to adalimumab are associated with future inflammation in Crohn's patients receiving maintenance adalimumab therapy: a *post hoc* analysis of the Karmiris trial. Gut 65 (7), 1126–1131. 10.1136/gutjnl-2014-307882 25862647

[B3] Bartelds G. M. Krieckaert C. L. Nurmohamed M. T. van Schouwenburg P. A. Lems W. F. Twisk J. W. (2011). Development of antidrug antibodies against adalimumab and association with disease activity and treatment failure during long-term follow-up. JAMA 305 (14), 1460–1468. 10.1001/jama.2011.406 21486979

[B4] Bartelds G. M. Wijbrandts C. A. Nurmohamed M. T. Stapel S. Lems W. F. Aarden L. (2010). Anti-infliximab and anti-adalimumab antibodies in relation to response to adalimumab in infliximab switchers and anti-tumour necrosis factor naive patients: a cohort study. Ann. Rheum. Dis. 69 (5), 817–821. 10.1136/ard.2009.112847 19581278

[B5] Battat R. Hemperly A. Truong S. Whitmire N. Boland B. S. Dulai P. S. (2021). Baseline clearance of infliximab is associated with requirement for colectomy in patients with acute severe ulcerative colitis. Clin. Gastroenterol. Hepatol. 19 (3), 511–518.e6. 10.1016/j.cgh.2020.03.072 32348905 PMC7606215

[B6] Brandse J. F. Mathot R. A. van der Kleij D. Rispens T. Ashruf Y. Jansen J. M. (2016). Pharmacokinetic features and presence of antidrug antibodies associate with response to infliximab induction therapy in patients with moderate to severe ulcerative colitis. Clin. Gastroenterol. Hepatol. 14 (2), 251–258. 10.1016/j.cgh.2015.10.029 26545802

[B7] Brandse J. F. van den Brink G. R. Wildenberg M. E. van der Kleij D. Rispens T. Jansen J. M. (2015). Loss of infliximab into feces is associated with lack of response to therapy in patients with severe ulcerative colitis. Gastroenterology 149 (2), 350–355. 10.1053/j.gastro.2015.04.016 25917786

[B8] Cheifetz A. S. Abreu M. T. Afif W. Cross R. K. Dubinsky M. C. Loftus E. V. Jr. (2021). A comprehensive literature review and expert consensus statement on therapeutic drug monitoring of biologics in inflammatory bowel disease. Am. J. Gastroenterol. 116 (10), 2014–2025. 10.14309/ajg.0000000000001396 34388143 PMC9674375

[B9] Chen D. Y. Chen Y. M. Hsieh T. Y. Hung W. T. Hsieh C. W. Chen H. H. (2016). Drug trough levels predict therapeutic responses to dose reduction of adalimumab for rheumatoid arthritis patients during 24 weeks of follow-up. Rheumatol. Oxf. 55 (1), 143–148. 10.1093/rheumatology/kev298 26324949

[B10] Colombel J. F. Sandborn W. J. Rutgeerts P. Enns R. Hanauer S. B. Panaccione R. (2007). Adalimumab for maintenance of clinical response and remission in patients with Crohn's disease: the CHARM trial. Gastroenterology 132 (1), 52–65. 10.1053/j.gastro.2006.11.041 17241859

[B11] D’Haens G. R. Sandborn W. J. Loftus E. V. Hanauer S. B. Schreiber S. Peyrin-Biroulet L. (2022). Higher vs standard adalimumab induction dosing regimens and two maintenance strategies: randomized SERENE CD trial results. Gastroenterology 162 (7), 1876–1890. 10.1053/j.gastro.2022.01.044 35122766

[B12] Ding X. Zhu R. Wu J. Xue L. Gu M. Miao L. (2020). Early adalimumab and anti-adalimumab antibody levels for prediction of primary nonresponse in ankylosing spondylitis patients. Clin. Transl. Sci. 13 (3), 547–554. 10.1111/cts.12738 31961477 PMC7214645

[B13] Fautrel B. Pham T. Alfaiate T. Gandjbakhch F. Foltz V. Morel J. (2016). Step-down strategy of spacing TNF-blocker injections for established rheumatoid arthritis in remission: results of the multicentre non-inferiority randomised open-label controlled trial (STRASS: spacing of TNF-blocker injections in Rheumatoid ArthritiS Study). Ann. Rheum. Dis. 75 (1), 59–67. 10.1136/annrheumdis-2014-206696 26103979

[B14] Feuerstein J. D. Nguyen G. C. Kupfer S. S. Falck-Ytter Y. Singh S. American Gastroenterological Association Institute Clinical Guidelines C (2017). American gastroenterological association institute guideline on therapeutic drug monitoring in inflammatory bowel disease. Gastroenterology 153 (3), 827–834. 10.1053/j.gastro.2017.07.032 28780013

[B15] Irving P. M. Gecse K. B. (2022). Optimizing therapies using therapeutic drug monitoring: current strategies and future perspectives. Gastroenterology 162 (5), 1512–1524. 10.1053/j.gastro.2022.02.014 35167865

[B16] Jamnitski A. Bartelds G. M. Nurmohamed M. T. van Schouwenburg P. A. van Schaardenburg D. Stapel S. O. (2011). The presence or absence of antibodies to infliximab or adalimumab determines the outcome of switching to etanercept. Ann. Rheum. Dis. 70 (2), 284–288. 10.1136/ard.2010.135111 21068090

[B17] Jongsma M. M. E. Winter D. A. Huynh H. Q. Norsa L. Hussey S. Kolho K. L. (2020). Infliximab in young paediatric IBD patients: it is all about the dosing. Eur. J. Pediatr. 179 (12), 1935–1944. 10.1007/s00431-020-03750-0 32813123 PMC7666662

[B18] Khan A. Berahmana A. B. Day A. S. Barclay M. L. Schultz M. (2019). New Zealand society of gastroenterology guidelines on therapeutic drug monitoring in inflammatory bowel disease. N. Z. Med. J. 132 (1491), 46–62. 30845128

[B19] Krieckaert C. L. Nair S. C. Nurmohamed M. T. van Dongen C. J. Lems W. F. Lafeber F. P. (2015). Personalised treatment using serum drug levels of adalimumab in patients with rheumatoid arthritis: an evaluation of costs and effects. Ann. Rheum. Dis. 74 (2), 361–368. 10.1136/annrheumdis-2013-204101 24265411

[B20] Krieckaert C. L. van Tubergen A. Gehin J. E. Hernandez-Breijo B. Le Meledo G. Balsa A. (2023). EULAR points to consider for therapeutic drug monitoring of biopharmaceuticals in inflammatory rheumatic and musculoskeletal diseases. Ann. Rheum. Dis. 82 (1), 65–73. 10.1136/annrheumdis-2022-222155 35551063

[B21] Lamers-Karnebeek F. B. G. Jacobs J. W. G. Radstake T. van Riel P. Jansen T. L. (2019). Adalimumab drug and antidrug antibody levels do not predict flare risk after stopping adalimumab in RA patients with low disease activity. Rheumatol. Oxf. 58 (3), 427–431. 10.1093/rheumatology/key292 30383251

[B22] l’Ami M. J. Krieckaert C. L. Nurmohamed M. T. van Vollenhoven R. F. Rispens T. Boers M. (2018). Successful reduction of overexposure in patients with rheumatoid arthritis with high serum adalimumab concentrations: an open-label, non-inferiority, randomised clinical trial. Ann. Rheum. Dis. 77 (4), 484–487. 10.1136/annrheumdis-2017-211781 28939629

[B23] Mitrev N. Vande Casteele N. Seow C. H. Andrews J. M. Connor S. J. Moore G. T. (2017). Review article: consensus statements on therapeutic drug monitoring of anti-tumour necrosis factor therapy in inflammatory bowel diseases. Aliment. Pharmacol. Ther. 46 (11-12), 1037–1053. 10.1111/apt.14368 29027257

[B24] Nguyen N. H. Solitano V. Vuyyuru S. K. MacDonald J. K. Syversen S. W. Jorgensen K. K. (2022). Proactive therapeutic drug monitoring versus conventional management for inflammatory bowel diseases: a systematic review and meta-analysis. Gastroenterology 163 (4), 937–949.e2. 10.1053/j.gastro.2022.06.052 35753383

[B25] Page M. J. McKenzie J. E. Bossuyt P. M. Boutron I. Hoffmann T. C. Mulrow C. D. (2021). The PRISMA 2020 statement: an updated guideline for reporting systematic reviews. BMJ 372, n71. 10.1136/bmj.n71 33782057 PMC8005924

[B26] Panes J. Colombel J. F. D'Haens G. R. Schreiber S. Panaccione R. Peyrin-Biroulet L. (2022). Higher vs standard adalimumab induction and maintenance dosing regimens for treatment of ulcerative colitis: SERENE UC trial results. Gastroenterology 162 (7), 1891–1910. 10.1053/j.gastro.2022.02.033 35227777

[B27] Papamichael K. Afif W. Drobne D. Dubinsky M. C. Ferrante M. Irving P. M. (2022). Therapeutic drug monitoring of biologics in inflammatory bowel disease: unmet needs and future perspectives. Lancet Gastroenterol. Hepatol. 7 (2), 171–185. 10.1016/S2468-1253(21)00223-5 35026171 PMC10187071

[B28] Papamichael K. Juncadella A. Wong D. Rakowsky S. Sattler L. A. Campbell J. P. (2019). Proactive therapeutic drug monitoring of adalimumab is associated with better long-term outcomes compared with standard of care in patients with inflammatory bowel disease. J. Crohns Colitis 13 (8), 976–981. 10.1093/ecco-jcc/jjz018 30689771 PMC6939875

[B29] Papamichael K. Rakowsky S. Rivera C. Cheifetz A. S. Osterman M. T. (2018). Infliximab trough concentrations during maintenance therapy are associated with endoscopic and histologic healing in ulcerative colitis. Aliment. Pharmacol. Ther. 47 (4), 478–484. 10.1111/apt.14458 29210094 PMC6535226

[B30] Plasencia C. Pascual-Salcedo D. Garcia-Carazo S. Lojo L. Nuno L. Villalba A. (2013). The immunogenicity to the first anti-TNF therapy determines the outcome of switching to a second anti-TNF therapy in spondyloarthritis patients. Arthritis Res. Ther. 15 (4), R79. 10.1186/ar4258 23890223 PMC3978754

[B31] Pouw M. F. Krieckaert C. L. Nurmohamed M. T. van der Kleij D. Aarden L. Rispens T. (2015). Key findings towards optimising adalimumab treatment: the concentration-effect curve. Ann. Rheum. Dis. 74 (3), 513–518. 10.1136/annrheumdis-2013-204172 24326008

[B32] Rau R. (2002). Adalimumab (a fully human anti-tumour necrosis factor alpha monoclonal antibody) in the treatment of active rheumatoid arthritis: the initial results of five trials. Ann. Rheum. Dis. 61 (Suppl. 2), ii70–3. 10.1136/ard.61.suppl_2.ii70 12379628 PMC1766697

[B33] Rinawi F. Ricciuto A. Church P. C. Frost K. Crowley E. Walters T. D. (2021). Association of early postinduction adalimumab exposure with subsequent clinical and biomarker remission in children with Crohn's disease. Inflamm. Bowel Dis. 27 (7), 1079–1087. 10.1093/ibd/izaa247 32978946

[B34] Roblin X. Genin C. Nancey S. Williet N. Veyrard P. Boschetti G. (2022). Swapping versus dose optimization in patients losing response to adalimumab with adequate drug levels. Inflamm. Bowel Dis. 28 (5), 720–727. 10.1093/ibd/izab158 34405867

[B35] Roblin X. Rinaudo M. Del Tedesco E. Phelip J. M. Genin C. Peyrin-Biroulet L. (2014). Development of an algorithm incorporating pharmacokinetics of adalimumab in inflammatory bowel diseases. Am. J. Gastroenterol. 109 (8), 1250–1256. 10.1038/ajg.2014.146 24913041

[B36] Stang A. (2010). Critical evaluation of the Newcastle-Ottawa scale for the assessment of the quality of nonrandomized studies in meta-analyses. Eur. J. Epidemiol. 25 (9), 603–605. 10.1007/s10654-010-9491-z 20652370

[B37] Sterne J. A. C. Savovic J. Page M. J. Elbers R. G. Blencowe N. S. Boutron I. (2019). RoB 2: a revised tool for assessing risk of bias in randomised trials. BMJ 366, l4898. 10.1136/bmj.l4898 31462531

[B38] Tweehuysen L. van den Ende C. H. Beeren F. M. Been E. M. van den Hoogen F. H. den Broeder A. A. (2017). Little evidence for usefulness of biomarkers for predicting successful dose reduction or discontinuation of a biologic agent in rheumatoid arthritis: a systematic review. Arthritis Rheumatol. 69 (2), 301–308. 10.1002/art.39946 27696778 PMC5299504

[B39] Ulijn E. den Broeder N. Wientjes M. van Herwaarden N. Meek I. Tweehuysen L. (2020). Therapeutic drug monitoring of adalimumab in RA: no predictive value of adalimumab serum levels and anti-adalimumab antibodies for prediction of response to the next bDMARD. Ann. Rheum. Dis. 79 (7), 867–873. 10.1136/annrheumdis-2020-216996 32317314

[B40] Ungar B. Mazor Y. Weisshof R. Yanai H. Ron Y. Goren I. (2016). Induction infliximab levels among patients with acute severe ulcerative colitis compared with patients with moderately severe ulcerative colitis. Aliment. Pharmacol. Ther. 43 (12), 1293–1299. 10.1111/apt.13631 27091119

[B41] van Herwaarden N. van der Maas A. Minten M. J. van den Hoogen F. H. Kievit W. van Vollenhoven R. F. (2015). Disease activity guided dose reduction and withdrawal of adalimumab or etanercept compared with usual care in rheumatoid arthritis: open label, randomised controlled, non-inferiority trial. BMJ 350, h1389. 10.1136/bmj.h1389 25858265 PMC4391970

[B42] Ward M. G. Warner B. Unsworth N. Chuah S. W. Brownclarke C. Shieh S. (2017). Infliximab and adalimumab drug levels in Crohn's disease: contrasting associations with disease activity and influencing factors. Aliment. Pharmacol. Ther. 46 (2), 150–161. 10.1111/apt.14124 28481014

[B43] Winter D. A. Joosse M. E. de Wildt S. N. Taminiau J. de Ridder L. Escher J. C. (2020). Pharmacokinetics, pharmacodynamics, and immunogenicity of infliximab in pediatric inflammatory bowel disease: a systematic review and revised dosing considerations. J. Pediatr. Gastroenterol. Nutr. 70 (6), 763–776. 10.1097/MPG.0000000000002631 32443029

[B44] Zittan E. Kabakchiev B. Milgrom R. Nguyen G. C. Croitoru K. Steinhart A. H. (2016). Higher adalimumab drug levels are associated with mucosal healing in patients with Crohn's disease. J. Crohns Colitis 10 (5), 510–515. 10.1093/ecco-jcc/jjw014 26783345 PMC4957459

