# Supplementary material for: What are the benefits of therapeutic drug monitoring in the optimization of adalimumab therapy? a systematic review and meta-analysis up to 2022
Source: Front Pharmacol. 2024 Jul 8;15:1376708. doi: 10.3389/fphar.2024.1376708 (PMC11260779; doi:10.3389/fphar.2024.1376708)
Supplement: Supplementary file 1 [file Table1.DOCX]

Supplementary Table 1 Specific search history for the three databases.

| **number** | **Query** |
| --- | --- |
| **PubMed** | |
| 15 | (((adalimumab[MeSH Terms]) OR (adalimumab[Title/Abstract])) OR (humira[Title/Abstract])) AND (((((((((drug monitoring[MeSH Terms]) OR (tdm[Title/Abstract])) OR ("therapeutic monitor*"[Title/Abstract])) OR ("drug monitor*"[Title/Abstract])) OR (anti?drug*[Title/Abstract])) OR (anti?adalimumab[Title/Abstract])) OR (anti?humira[Title/Abstract])) OR (concentration*[Title/Abstract])) OR (trough[Title/Abstract])) |
| 14 | ((((((((drug monitoring[MeSH Terms]) OR (tdm[Title/Abstract])) OR ("therapeutic monitor*"[Title/Abstract])) OR ("drug monitor*"[Title/Abstract])) OR (anti?drug*[Title/Abstract])) OR (anti?adalimumab[Title/Abstract])) OR (anti?humira[Title/Abstract])) OR (concentration*[Title/Abstract])) OR (trough[Title/Abstract]) |
| 13 | trough[Title/Abstract] |
| 12 | concentration*[Title/Abstract] |
| 11 | anti?humira[Title/Abstract] |
| 10 | anti?adalimumab[Title/Abstract] |
| 9 | anti?drug*[Title/Abstract] |
| 8 | "drug monitor*"[Title/Abstract] |
| 7 | "therapeutic monitor*"[Title/Abstract] |
| 6 | tdm[Title/Abstract] |
| 5 | drug monitoring[MeSH Terms] |
| 4 | ((adalimumab[MeSH Terms]) OR (adalimumab[Title/Abstract])) OR (humira[Title/Abstract]) |
| 3 | humira[Title/Abstract] |
| 2 | adalimumab[Title/Abstract] |
| 1 | adalimumab[MeSH Terms] |
| **Embase** | |
| #1 | MeSH descriptor: [Adalimumab] explode all trees |
| #2 | (adalimumab):ti, ab, kw |
| #3 | (humira):ti, ab, kw |
| #4 | #1 OR #2 OR #3 |
| #5 | MeSH descriptor: [Drug Monitoring] explode all trees |
| #6 | (TDM):ti, ab, kw |
| #7 | ("therapeutic monitor*"):ti, ab, kw (Word variations have been searched) |
| #8 | ("drug monitor*"):ti, ab, kw (Word variations have been searched) |
| #9 | (anti?drug*):ti, ab, kw (Word variations have been searched) |
| #10 | (anti?adalimumab):ti, ab, kw (Word variations have been searched) |
| #11 | (anti?humira):ti, ab, kw (Word variations have been searched) |
| #12 | (concentration*):ti, ab, kw (Word variations have been searched) |
| #13 | (trough):ti, ab, kw |
| #14 | #5 OR #6 OR #7 OR #8 OR #9 OR #10 OR #11 OR #12 OR #13 |
| #15 | #4 AND #14 |
| **Cochrane library** | |
| #15 | #4 AND #14 |
| #14 | #5 OR #6 OR #7 OR #8 0R #9 0R#10 OR#11 0R #12 OR #13 |
| #13 | trough:ab, ti |
| #12 | concentration*:ab, ti |
| #11 | anti?humira:ab, ti |
| #10 | anti?adalimumab:ab, ti |
| #9 | anti?drug*:ab, ti |
| #8 | 'drug monitoring':ab, ti |
| #7 | 'therapeutic monitor*':ab, ti |
| #6 | tdm |
| #5 | 'drug monitoring'/exp |
| #4 | #1 OR #2 OR #3 |
| #3 | humira:ab, ti |
| #2 | adalimumab:ab, ti |
| #1 | 'adalimumab'/exp |
